# Severe acute hepatitis and acute liver failure of unknown origin in children: a questionnaire-based study within 34 paediatric liver centres in 22 European countries and Israel, April 2022

**DOI:** 10.2807/1560-7917.ES.2022.27.19.2200369

**Published:** 2022-05-12

**Authors:** Ruben H de Kleine, Willem S Lexmond, Gustav Buescher, Ekkehard Sturm, Deidre Kelly, Ansgar W Lohse, Dominic Lenz, Marianne Hørby Jørgensen, on behalf of the contributors to the survey

**Affiliations:** 1 Department of Surgery, Division of Hepatobiliairy Surgery and Liver Transplantation, University of Groningen, University Medical Centre Groningen, Groningen, The Netherlands; 2 European Reference Network for hepatological diseases (ERN RARE-LIVER); 3 Department of Paediatrics, Division of Gastroenterology and Hepatology, Beatrix Children’s Hospital, University of Groningen, Groningen, The Netherlands; 4 Department of Internal Medicine, University Medical Centre Hamburg-Eppendorf, Hamburg, Germany; 5 Paediatric Gastroenterology/ Hepatology, University Hospital Tuebingen, Tuebingen, Germany; 6 University of Birmingham, and The Liver Unit, Birmingham Women & Children’s Hospital, Birmingham, UK; 7 Division of Neuropaediatrics and Paediatric Metabolic Medicine, Centre for Paediatric and Adolescent Medicine, University Hospital Heidelberg, Heidelberg, Germany; 8 Department of Paediatric and Adolescent Medicine, Rigshospitalet, Copenhagen, Denmark; 9 The contributors to the survey are acknowledged at the end of the article

**Keywords:** paediatric, hepatitis, acute liver failure, liver transplantation, virus

## Abstract

To detect potential concern about severe acute hepatitis in children, we conducted a survey among 50 ERN RARE-LIVER centres. By 26 April 2022, 34 centres, including 25 transplant centres, reported an estimated median of 3–5, 0–2 and 3–5 cases in 2021, 2020 and 2019 and a mean of 2 (range: 0–8) cases between January and April 2022 (mean in 10 large liver transplant centres: 3). Twelve centres reported suspicion of an increase, but no rise.

Following a report by the United Kingdom (UK) on 5 April 2022 on the occurrence of cases of severe acute hepatitis in children aged 16 years or under, the World Health Organization (WHO) raised concerns about the possibility of an epidemic [1,2]. By 21 April, 169 possible or confirmed cases were reported fulfilling the WHO case definition [3]. The cause of the hepatitis is unknown but a link to a virus infection has been suggested due to the epidemiological pattern of cases [4,5]. The hepatitis can progress to paediatric acute liver failure (pALF) necessitating urgent liver transplantation to avoid multi-organ failure [6]. We intended to assess whether a rise in incidence of severe acute hepatitis or pALF could be observed between 1 January and 26 April 2022 in comparison to previous years, within the European Reference Network on Hepatological Diseases (ERN RARE-LIVER) [7].

## Questionnaire survey

The web-based questionnaire survey available from the Supplementary material was sent to 50 (associate) member centres of the ERN RARE-LIVER involved in the treatment of children with hepatitis. Using the European Union (EU) Survey tool, the survey started on 15 April and was open for responses until 26 April. Data retrieved were managed from the secure EU Survey tool. Contributors were asked to register their name and email before entering anonymous data from their own case-defined patients at their centre. After the upload, a summary of their data was sent back for verification via email.

The survey questionnaire was divided into two parts with the first part related to the incidence and treatment of paediatric hepatitis and pALF patients fulfilling the definition of the Paediatric Acute Liver Failure Study Group (Box), in the 3 preceding years [8]. Participants were asked to provide an estimate with the options 0─2, 3─5, 6─10, 11─20 patients.

Box **Case definition of severe non-A-E hepatitis and definition of paediatric acute liver failure**

Severe non-A-E hepatitis

- Rise in aspartate-aminotransferase (AST) or alanine-aminotransferase (ALT) of more than 500 units per litre (U/L).
- Age ≤ 16 years.
- Since 1 January 2022.
- No A─E hepatitis virus detected.

***Paediatric acute liver failure***

- Evidence of acute hepatic injury (elevated ALT).
- Spontaneous INR ≥ 2.0.
- Or INR ≥ 1.5 with signs of hepatic encephalopathy.

INR: international normalised ratio.

Questions in the second part of the survey focussed on patients seen in 2022 in line with the WHO definition of 15 April 2022 [1]. Responses captured anonymous details on all children admitted with severe hepatitis or pALF (Box) and data on demographics (age, sex, aetiology), clinical course (cause identified, possible cause, indetermined) and outcome (survival with native liver, liver transplantation indicated, overall survival) were entered.

This rapid communication was written with STROBE criteria in consideration [9].

## Participating centres and responses

The survey was completed by 34 centres from 22 countries of which 31 are (associate) members of the ERN RARE-LIVER. This network is dedicated to the improvement of care for rare liver diseases by collaboration through innovation, research and exchange of data for all involved patients [7]. The location of participating centres is mapped in the Figure.

**Figure:**
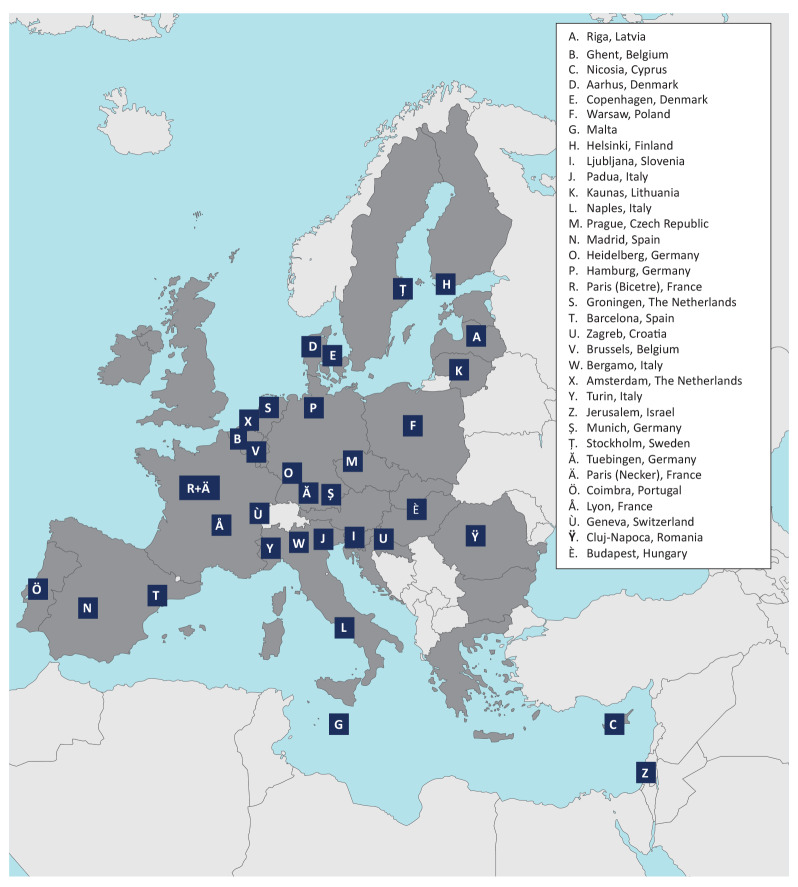
Location of the centres participating in the survey (n = 34)

All centres treat children with hepatitis, while 25 of the responding 34 centres also perform paediatric liver transplantation. Except for contributing centres in Switzerland and Israel, all participants were from the EU. No data from the UK were entered in the query.

## Incidence of severe acute hepatitis or pALF

Of the 34 participating centres, 22 reported no suspicion of an increase of children with severe hepatitis between January and April 2022, with 10 centres reporting no new paediatric hepatitis patients in this period. All centres reported seeing patients with hepatitis in all previous 3 years with further details listed in Table 1.

**Table 1:** Centres’ reported estimated numbers of children with severe hepatitis or paediatric acute liver failure, European Reference Network Rare Liver survey, 1 January 2019–26 April 2022 (n = 34)

Number of children with pALF [8] per centre	2019 Number of centres	2020 Number of centres	2021 Number of centres	2022^a^ Number of centres (exact number of cases)
0─2	16	18	5	11 (0)
				6 (1)
				7 (2)
3─5	10	9	9	3 (3)
				2 (4)
				3 (5)
6─10	8	7	8	1 (8)
11─20	0	0	2	0

pALF: paediatric acute liver failure.

^a^ Data cover only 3.8 months and comprises pALF and acute severe hepatitis.

In the study period of January to April 2022, a mean of 2 (range: 0–8) cases was detected. Twelve centres reported a suspected increase but documented no rise in numbers. The 11 large transplant centres with 16 or more paediatric liver transplantations per year, reported a mean of 2.5 cases (range: 0─5) referred for transplantation in the 3.8 months of 2022, while in 2019, 2020 and 2021 on average 4.9 (range: 0─10), 3.7 (range: 0─10) and 4.9 (range: 0─10) cases per year were reported, respectively.

## Details of children with severe hepatitis and paediatric acute liver failure in 2022

We received detailed information on 65 children with severe hepatitis (n = 59) or pALF (n = 33, 4 missing data, INR ≥2.0) treated at 19 different centres. One was excluded since they were over 16 years of age. Four of 64 children did not fulfil the criteria for severe hepatitis but were included since they presented with the more severe condition of pALF. Therefore, some groups show overlap in numbers.

An extensive diagnostic work-up was documented in all children (Table 2). In 11 children the cause for the clinical condition was identified, while the cause remained indeterminate for 27 children (26 severe hepatitis and 10 pALF, with one child in the pALF group not fulfilling the hepatitis criteria). The centres reported a possible cause of the severe hepatitis or pALF episode without final certainty in 26 children (24 severe hepatitis; 17 pALF, with two children in the pALF group not fulfilling the hepatitis criteria).

**Table 2:** Characteristics of children with severe hepatitis or paediatric acute liver failure by cause, ERN RARE-LIVER survey, 1 January–26 April 2022 (n = 64)

Baseline characteristics	All children (mean, range) n = 64	Cause identified (mean, range) n = 11	Possible cause identified (mean, range) n = 27	No cause identified (mean, range) n = 26
Sex female vs male	28 vs 35 (1 missing)	5 vs 6	17 vs 9 (1 missing)	13 vs 13
Age	7.7 years (28 days–16 years)	5.5 years (28 days–16 years)	5.8 years (66 days–16 years) (2 missing)	5.8 years (56 days–16 years
Admission to clinic	64	11	27	26
Laboratory findings				
ALT (U/L)^a^	2,871 (90–16,686)	1,115 (90–6,350)	3,640 (100–16,686)	2,736 (515–9,000)
Bilirubin (µmol/L)^a^	100 (0–470)	106 (1–534)	107 (0–470)	88 (4–468)
INR^a^	2.98 (0.89–16.70)	3.18 (1.13–7.00)	3.49 (1.00–16.70)	2.29 (0.96–6.70)
NH_3_	104 (26–472)	95 (45–215)	105 (26–266)	108 (27–472)
Viruses detected	13	2 (enterovirus, EBV)	9 patients, multiple viruses (4 adenovirus, 4 SARS-CoV-2, 1 rotavirus, 1 influenza A virus, 2 EBV)^b^	2 (rotavirus, influenza A virus)
Active SARS-CoV-2 infection at admission (n, %)	3 (5%)	0	3 (11%)	0
SARS-CoV-2 vaccinated (n, %)	5 (3 missing data) (8%)	0 (2 missing data)	4 (1 missing data)	1

EBV: Epstein-Barr virus; INR: international normalised ratio; NH_3_: ammonia; SARS-CoV-2: severe acute respiratory syndrome coronavirus 2.

^a^ Highest value selected.

^b^ Co-infection in one patient with rotavirus, adenovirus and SARS-CoV-2. Another patient with EBV and adenovirus.

A previously known cause for severe acute hepatitis in children, such as an underlying medical condition, was present in 16 of 64 children, comprising congenital defects (n = 4), immune deficiency (n = 1), metabolic disorders (n = 3) as well a wide range of other disease like a structural heart defect, bone marrow disease, immune therapy for malignancy or structural defects otherwise unclassified. Different viruses were detected in 13 children with adenovirus being present in four (Table 2). Five of 64 children (aged 7 years or older) were vaccinated against coronavirus disease (COVID-19). These five received the vaccination at least 4 weeks before the onset of symptoms of hepatitis with no report of adverse effects or complaints in between. No link between the vaccination and the liver disease was suspected by any of the contributors.

In total four children received a liver transplant, three died before an organ was available. One child with Wilson’s disease (cause identified) died after transplantation (Table 3).

**Table 3:** Outcome of children with severe acute hepatitis or paediatric acute liver failure by cause identified ERN RARE-LIVER survey, 1 January–26 April 2022 (n = 64)

Outcome as at 26 April	All children (mean, range) n = 64 (4 missing data)	Cause identified n = 11	Possible cause identified (mean, range) n = 27 (2 missing data)	No cause identified n = 26 (2 missing data)
Ongoing hepatitis	9	0	5	4
Survival with native liver	43	8	17	18
LTX (n)	4	1	1	2
Deceased before/after LTX (n)	3/1	2/1	1/0	0/0

LTX: liver transplantation.

## Discussion

We present results from a European survey among specialised liver centres across Europe and in Israel to provide further insight for the recently reported increase in severe acute hepatitis cases in children. Compared with the average of cases in each of the full previous years 2019-21, there was no absolute increase of cases within stated criteria in the study period, based on the data from the contributing centres. However, the data for 2022 comprise only the first 3.8 months of the year and should be considered preliminary. Extrapolation of these data into the future is tempting but should be done with caution.

While existing data on the hepatitis increase in children the UK and the United States suggest a role for adenovirus infections [1,10], we did not detect adenoviruses in the majority of patients, nor another uniform viral infection. Interestingly, severe hepatitis with abdominal symptoms suggestive of a gastrointestinal virus, was reported in 1923 [11] following the 1918 influenza pandemic. It was then considered to be related to susceptibility to viruses people had not been exposed to during social containment. This parallelism supports a possible role for viral pathogens in the current situation. The exact pathomechanism causing hepatitis remains largely unknown, however, an interaction between the immature or naïve immune system and the liver might play an important role. Based on the portal vein blood flow, directly coming from the intestine, and the described clinical presentation of abdominal pain, illness and vomiting [1], such a trigger might be coming from an infection with one of the enteric viruses, such as adenovirus type 41 and 42.

Our study is limited by the format of a self-reported questionnaire which comes with its own form of reporting bias. Sixteen of the 50 approached centres did not respond. This may have led to an overestimation of the actual numbers within the network since the mean number was calculated only from responders. Absolute numbers of paediatric severe hepatitis remain low and numbers of pALF have been even lower in 2022 and previously. Therefore, formal conclusions are difficult to make. Our survey does not give additional information to prompt extra caution or specific protective measures at this very moment. This variation of hepatitis and pALF occurrence needs further investigation and could benefit from a pan-European collaboration to research and biobank material for modern viral detection techniques in order to identify the underlying cause. Awareness among paediatric hepatologists about a possible rise in hepatitis is high and this should be a good basis for establishing a reliable platform for cutting edge research.

### Conclusion

Currently, there is no clear overall increase in the occurrence of severe hepatitis or pALF in children in Europe, when we compare the reported data of previous years to the preliminary data from the study period of 3.8 months in 2022. By 26 April, only a minority of the centres within the ERN RARE-LIVER appear to be affected by a possible rise in hepatitis cases. No increase in virus-derived hepatitis could be detected at this point in time within the whole group of participating centres. However, a close monitoring will be necessary in order to achieve early signal detection of this clinical condition.

## Supplementary Data

123000 Supplementary Material
