# Supplementary material for: Severe acute hepatitis and acute liver failure of unknown origin in children: a questionnaire-based study within 34 paediatric liver centres in 22 European countries and Israel, April 2022
Source: Euro Surveill. 2022 May 12;27(19):2200369. doi: 10.2807/1560-7917.ES.2022.27.19.2200369 (PMC9101968; doi:10.2807/1560-7917.ES.2022.27.19.2200369)
Supplement: Supplementary Material [file 22-00369_DE_KLEINE_Supplementary_material.pdf]

This supplementary material is hosted by *Eurosurveillance* as supporting information alongside the article 'Severe acute hepatitis and acute liver failure of unknown origin in children: a questionnaire-based study within 34 paediatric liver centres in 22 European countries and Israel, April 2022', on behalf of the authors, who remain responsible for the accuracy and appropriateness of the content. The same standards for ethics, copyright, attributions and permissions as for the article apply. Supplements are not edited by *Eurosurveillance* and the journal is not responsible for the maintenance of any links or email addresses provided therein.

European Reference Network
for rare or low prevalence complex diseases

Network
Hepatological Diseases
(ERN RARE-LIVER)

Dear Member of the ERN Rare Liver Network,

On behave of the Acute Liver Working group; we would like to draw your attention to the following: Some of our members have noticed a possible rise in the number of acute hepatitis in children with several of these patients needing specialized care. An official warning (https://www.ecdc.europa.eu/en/news-events/increase-acute-hepatitis-unknown-origin-among-children-united-kingdom) has been issued for the UK by the EDC supported by a Scottish publication (https://www.eurosurveillance.org/content/10.2807/1560-7917.ES.2022.27.15.2200318). We would like to know if your centre has encountered more than normal number of children with hepatitis, possibly leading to a higher rate of acute liver failure. If you are physician working in the field of paediatric hepatology please fill in this questionnaire urgently. If this is not your area of expertise, please forward this request to your paediatric department. If this query does not open due to technical problems please try to copy the link into another browser (i.e. firefox).

Sincerely, Ruben de Kleine

ERN Project leader Covid and non-covid related to paediatric Acute Liver Failure (CALF)
Surgeon at the UMCG, Groningen, The Netherlands

Members

D. Lenz, Chair Acute Liver Failure working Group, Universitätsklinikum Heidelberg, Heidelberg, Germany

W. Lexmond, paediatric hepatologist, University Medical Centre Groningen, Groningen, The Netherlands

M. Hørby Jørgensen, paediatric hepatologist, Rigshospitalet Copenhagen, Copenhagen, Denmark

E. Sturm, paediatric hepatologist, Universitätsklinikum Tübingen, Tübingen, Germany

D. Kelly, paediatric hepatologist, University of Birmingham, Birmingham, UK

M. Faber, Senior Researcher, Robert Koch Institute, Berlin, Germany

A. Lohse, President ERN Rare Liver, Hamburg, Germany

Fields marked with * are mandatory.

Questionnaire Covid-Non covid related to paediatric Acute Liver Failure (CALF query)
This questionnaire will take approximately 4 minutes. Per ERN centre one author will be added to the contributor group per 5 patients entered in the second part of the query. Data is managed through the ERN agreement and processed anonymously by the ERN. Data remains the property of the ERN member hospital. If you want to include more than 5 patients, please submit and complete a new survey (this allows a further contributer of your center to be included).

* Name of contributer

* Email of contributer

* Center of contributer

* Country of contributer

Are you a (associate) Member of ERN Rare Liver?

- (x) yes
- ( ) no

* Do you treat children with acute liver failure/non A-E hepatitis?

- [x] yes
- [ ] no

* Do you transplant children at your center?

- [x] yes
- [ ] no

On average how many children do you transplant per year at your centre?

- [ ] 1-5
- [ ] 6-10
- [ ] 11-15
- [ ] 16-20
- [ ] 21-25
- [ ] >25

Number of patients at your center with pALF (INR> 2.0):

* 2019:

- [ ] 0-2
- [ ] 3-5
- [ ] 6-10
- [ ] 11-20

* 2020:

- [ ] 0-2
- [ ] 3-5
- [ ] 6-10
- [ ] 11-20

* 2021:

- [ ] 0-2
- [ ] 3-5
- [ ] 6-10
- [ ] 11-20

* Jan 2022 – April 15 (actual number):

Only values of at least 0 are allowed

* Has there been a suspicion of an increase in the number of children with pALF (ALT elevation and INR>2) or severe hepatitis (ALT>500) at your center during the last 4 months (Jan 1 - April 14 2022)?

- [x] yes
- [ ] no

* Are you able to provide data on these patients (year 2022) for clinical purposes to ascertain whether there is a possible emergency?

- (x) yes
- ( ) no

Name Physician (for inclusion in the contributors group)

Patient query. This part will take 4 minutes per patient.

Patient 1

Date of Birth (Month/Year)

Sex

- ( ) Female
- ( ) Male

Past medical history

- ( ) None
- ( ) Immune deficiency
- ( ) Metabolic disorder
- ( ) Congenital defect
- (x) Other

Please specify 'Other Past Medical history'

Medication use:

At time of first laboratory investigations that indicated liver disease, was there a recent history of (check all that apply):

at most 9 choice(s)

- [ ] Vomiting
- [ ] Diarrhoea
- [ ] Anorexia
- [ ] Fever
- [ ] Upper respiratory tract infection

- [ ] Jaundice
- [ ] Itching
- [ ] Acholic stools
- [ ] Drowsiness or mental status abnormalities

Biochemistry (please record the most abnormal (peak) values over the course of disease)

ALT

[ ] U/l

INR

[ ]

Total bilirubin

[ ] µmol/l

Direct bilirubin

[ ] µmol/l

NH_3

[ ] µmol/l

Factor V

[ ] % of normal

Medical course (please select all that apply):

between 1 and 4 choices

- [ ] Managed as an outpatient
- [ ] Admitted to non-transplant hospital
- [ ] Admitted to transplant center: patient ward
- [ ] Admitted to transplant center: PICU

Outcome

between 1 and 4 choices

- [ ] Resolved with native liver
- [ ] Ongoing hepatitis/liver failure
- [ ] Emergency liver transplantation
- [ ] Death before liver transplantation
- [ ] Death despite liver transplantation

Etiology

at least 1 choice(s)

- [x] Cause identified
- [x] Possible cause identified
- [x] No cause identified

Cause

[ ]

Possible cause

[ ]

If no cause identified, which etiologies have been ruled out (please check all that apply):

- [ ] Hepatitis A
- [ ] Hepatitis B
- [ ] Hepatitis C
- [ ] Hepatitis D
- [ ] Hepatitis E
- [ ] Adenovirus
- [ ] Influenza A
- [ ] Enterovirus
- [ ] EBV
- [ ] CMV
- [ ] HSV 1/2
- [ ] VZV
- [ ] HHV-6
- [ ] HHV-7
- [ ] Parechovirus
- [ ] Parvo B19
- [ ] Toxicological screen including acetaminophen
- [x] Autoimmune
- [ ] Wilson disease
- [ ] HLH
- [ ] Vascular liver disease
- [ ] Tyrosinemia
- [ ] Galactosemia
- [ ] Urea Cycle Defects
- [ ] Fatty Acids Oxidation Disorders
- [ ] Mitochondrial disorders (via panel diagnostic/clinical exome)
- [ ] Other plausible genetic causes (e.g. NBAS, LARS1, ALDOB/HFI) via Exome Sequencing

Total IgG level

[ ] g/l

COVID-19 status

- (selected) Documented past infection
- Likely past exposure or infection but not tested (e.g. parents or siblings with documented COVID infection)
- No known past exposure or infection
- COVID-19 virus detectable at time of liver disease

Time between first positive test and onset of liver disease:

Vaccination against COVID-19

- no
- (selected) yes

Time between first vaccination and onset of liver disease

- < 7 days
- Between 1 and 4 weeks
- > 4 weeks

Did you observe signs of bone marrow failure?
(Platelets <50 x10e9/l and/or Neutrof. Gran <1.0x10e9/l)

- (selected) yes
- no

Platelets

x10e9/l

Neutr. granulocytes

x10e9/l

Do you want to add another patient?

- yes
- (selected) no

End of query. Thank you for your contribution to this ERN questionnaire. Feel free to contact the working group on pALF in case of questions regarding this project.

Contact

Contact Form
